# Interaction of Human Laminin Receptor with Sup35, the [*PSI* ^+^] Prion-Forming Protein from *S. cerevisiae*: A Yeast Model for Studies of LamR Interactions with Amyloidogenic Proteins

**DOI:** 10.1371/journal.pone.0086013

**Published:** 2014-01-08

**Authors:** Christine Pampeno, Irina L. Derkatch, Daniel Meruelo

**Affiliations:** 1 Gene Therapy Center, Cancer Institute and Department of Pathology, New York University School of Medicine, New York, New York, United States of America; 2 Department of Neuroscience, College of Physicians and Surgeons of Columbia University, New York, New York, United States of America; University of Maryland School of Medicine, United States of America

## Abstract

The laminin receptor (LamR) is a cell surface receptor for extracellular matrix laminin, whereas the same protein within the cell interacts with ribosomes, nuclear proteins and cytoskeletal fibers. LamR has been shown to be a receptor for several bacteria and viruses. Furthermore, LamR interacts with both cellular and infectious forms of the prion protein, PrP^C^ and PrP^Sc^. Indeed, LamR is a receptor for PrP^C^. Whether LamR interacts with PrP^Sc^ exclusively in a capacity of the PrP receptor, or LamR specifically recognizes prion determinants of PrP^Sc^, is unclear. In order to explore whether LamR has a propensity to interact with prions and amyloids, we examined LamR interaction with the yeast prion-forming protein, Sup35. Sup35 is a translation termination factor with no homology or functional relationship to PrP. Plasmids expressing LamR or LamR fused with the green fluorescent protein (GFP) were transformed into yeast strain variants differing by the presence or absence of the prion conformation of Sup35, respectively [*PSI* ^+^] and [*psi* ^−^]. Analyses by immunoprecipitation, centrifugal fractionation and fluorescent microscopy reveal interaction between LamR and Sup35 in [*PSI* ^+^] strains. The presence of [*PSI* ^+^] promotes LamR co-precipitation with Sup35 as well as LamR aggregation. In [*PSI* ^+^] cells, LamR tagged with GFP or mCherry forms bright fluorescent aggregates that co-localize with visible [*PSI* ^+^] foci. The yeast prion model will facilitate studying the interaction of LamR with amyloidogenic prions in a safe and easily manipulated system that may lead to a better understanding and treatment of amyloid diseases.

## Introduction

The laminin receptor-1 (LamR) is a multifunctional protein required for cell viability [1], [2], [3]. Originally isolated by its ability to bind laminin-1 [4], [5], [6], LamR has since been identified as a highly conserved ribosomal protein that has evolved extra-ribosomal functions in multicellular organisms [7].

As a cell surface receptor, LamR functions in cell migration [8], through interaction with and remodeling of the extracellular matrix [9]. In tumor cells these functions have been associated with increased invasiveness and metastasis [9], [10], [11], [12]. The role of LamR in cellular translation and proliferation [13] may account for the upregulation of LamR expression in tumor cells [10], [11]. LamR has also been identified as a receptor for bacterial [14], [15] and viral infections [16], [17], [18], [19]. For a review of LamR and associated pathologies see [20].

The LamR is also a receptor for cellular [21], [22] and infectious [23], [24] prion proteins, PrP^C^ and PrP^Sc^, respectively. The LamR was identified as an interacting partner with the human prion protein, PrP, in a yeast two-hybrid screen [22]. Function of LamR as a cell surface receptor for the cellular prion protein, PrP^C^, has been shown by co-localization of LamR and PrP^C^ on the surface of mouse neuronal cells, as well as by the ability of LamR antibody to block exogenous PrP^C^ cellular binding and subsequent internalization [21]. Physiological significance of LamR interaction with PrP^Sc^ has been demonstrated by the prevention of prion propagation in neuronal cells by incubation with LamR antibody or by transfection of LamR antisense RNA and siRNA [25]. Cultures of intestinal enterocytes have been shown to internalize bovine spongiform encephalopathy prions through binding to cell surface LamR [24], indicating that LamR may be involved in the initial stage of prion infections. Similarly, co-localization of LamR with scrapie and chronic wasting disease prions has also been demonstrated [23].

PrP^Sc^-associated prion disease belongs to the broader class of pathologies known as amyloidoses, among which are Alzheimer’s and Parkinson’s diseases. Prions propagate by conversion of soluble prionogenic PrP^C^ proteins into the aggregated PrP^Sc^ form in a concentration- and nucleation-dependent manner, similar to the process of amyloidosis. Upon conversion to the prion state, the proteins adopt a cross-beta-sheet-rich structure, typical of amyloids. Purified recombinant PrP proteins can polymerize to amyloid fibers, which are resistant to SDS denaturation and bind to the amyloid-binding fluorophore thioflavin T and Congo Red dye [26]. However, the relationship between PrP amyloid forming *in* *vitro*, and known as PrP^Res^, and prion infectivity is not completely defined. Infectious PrP^Sc^ and recombinant PrP^Res^ fibrils have been shown to differ structurally and to have different seeding specificities [27].

Starting from 1994, several prions and prion-like proteins have been identified in yeast *Saccharomyces cerevisiae* (reviewed in [28], [29]). While the physiological importance of the ability to form self-propagating structures is a subject of debate, the occurrence of prion proteins is evolutionally conserved, and, for several yeast prions, numerous prion-bearing strains were isolated from nature [29], [30], [31]. Prionogenic proteins do not share amino acid sequence or functional homology. The tendency to form amyloid structure appears to be dependent upon amino acid composition: abundance of polar residues and paucity of hydrophobic and charged residues [31]. Yeast prions show typical amyloidogenic properties (reviewed in [29], [32], [33]).

Sup35, a yeast prion-forming protein that has been extensively studied, is a translational termination factor (eRF3) in its soluble form [34], [35]. However, when aggregated as a prion [36], [37], [*PSI^+^*], Sup35 is unavailable to terminate protein synthesis. Under this condition, protein termination is suppressed as ribosomes occasionally read through stop codons. Introduction of a stop codon mutation within a gene encoding the metabolic enzyme, *ADE1* [38], engendered a model system for studying yeast prionogenesis. The model utilizes the different phenotypes of prion positive [*PSI* ^+^] *vs* prion negative [*psi* ^−^] strains [39], [40]. This model has already produced many valuable studies into the nature of prion propagation and amyloidogenesis [31], [41], [42], [43], [44]. Furthermore, a yeast [*PSI^+^*]-based model was used for drug-screening: compounds isolated for their ability to affect yeast prions in this system, have also been demonstrated to be effective against PrP prions in mammalian cell assays [45].

In this study, we utilize the yeast Sup35/[PSI+] prion model system to investigate the putative propensity of LamR to interact with prionogenic proteins by examining the association of LamR with non-PrP prions. Yeast plasmids expressing LamR were introduced into *Saccromyces cerivceae* [*PSI* ^+^] and [*psi* ^−^] strains. Evidence from immunoprecipitation, high-speed centrifugation assays and fluorescent microscopy reveal an interaction between LamR and Sup35 in [*PSI* ^+^] cells, indicating that LamR interacts with Sup35-based prion protein.

## Materials and Methods

### Yeast Strains

Yeast strains used are derivatives of 74-D694 *(MATa ade1-14 his3-* Δ*200 ura3-52 leu2-3, 112 trp1-289)* [*psi* ^−^][*PIN^+^*] [39]. [*PSI^+^*][*PIN^+^*] are strong and weak [*PSI* ^+^] isolates (L1762 and L1759, respectively) obtained by overexpression of the Sup35 prion domain [40]. Isolates [*psi* ^−^][*pin* ^−^] (L1951) and strong (L1763) and weak (L1759) [*PSI^+^*][*pin* ^−^] were obtained from [*psi* ^−^][*PIN^+^*] (L1749), strong [*PSI^+^*][*PIN^+^*] (L1762) and weak [*PSI^+^*][*PIN^+^*] (1758), respectively, by curing [*PIN^+^*] upon growing yeast on media containing 5 mM GuHCl [46], [47].

### Plasmids and Transformation

The centromeric pRS400 series plasmids are the backbone for plasmids used in this study [48]. The *URA3* pRS416-based pCUP1-GFP encodes GFP expressed under the control of the copper inducible *CUP1* promoter, and pCUP1-SUP35::GFP encodes the *SUP35* ORF fused to the N-terminus of GFP [49]. These plasmids were a gift from S. Lindquist to I. Derkatch.

PCR fragments, synthesized from a human LamR expression vector [50], were generated to construct pCUP1-LamR and pCUP1-LamR::GFP; 5′ primer: *BamHI GGATCC* ATGTCCGGAGCCCTTGATGTCC; 3′ primers: *SacII CCGCGG* TTAAGACCAGTCAGTGGTTGCTCC with a stop codon at the end of the LamR ORF, and *SacII CCGCGG* AGACCAGTCAGTGGTTGCTCCTAC for LamR::GFP. Fragments were inserted into the pCUP1-GFP plasmid (see above).

To co-express the *URA3* pCUP1-SUP35::GFP with wild type or mCherry-tagged LamR, an *XhoI/SacII* fragment, containing the *CUP1* promoter and *LAMR*, was excised, from either the pRS416-pCUP1-LamR or the pRS416-pCUP1-LamR::GFP construct, and subcloned into the *LEU* plasmid, pRS415, to confer selective growth in leucineless medium. An mCherry-encoding PCR fragment was synthesized from a pmCherry vector (Clonetech, #632522) using primers: 5′ Primer: *SacII CCGCGG* ATGGTGAGCAAGGGC; 3′ primer *SacII CCGCGG* CTACAGCTCGTCCATGC. The *SacII/SacII* mCherry fragment was cloned into the *SacII* site of the construct with the *CUP1-LAMR* insert originating from pRS416-pCUP-LamR::GFP, to generate a LamR::mCherry fusion protein.

Standard yeast media and procedures were used [51]. Yeast transformants were grown in synthetic dextrose media selective for plasmid maintenance: SD-Ura, SD-Leu, or SD-Ura-Leu [51]. To induce the *CUP1* promoter, media were supplemented with 25 µM CuSO_4_.

### Yeast Lysates

Yeast cell lysates were prepared from mid-log cultures grown overnight at 30°C in plasmid-selective media supplemented with 25 µM CuSO_4_ (unless stated otherwise). Cells were harvested by centrifugation at 800×g for 10 min and washed with distilled H_2_O at RT. Pellets were resuspended in 2× volume lysis buffer [50 mM TrisHCl pH 7.5, 250 mM NaCl, 10 mM MgCl_2_, 5% w/v glycerol, anti-protease cocktail for yeast (Sigma) and 0.1 M AEBSF (Sigma)] and disrupted by beating with an equal volume of acid washed glass beads (425–600 µm, Sigma) [10 pulses of vortexing for 30 secs each, placing tubes on ice between vortexing to avoid heating]. Cell disruption was monitored microscopically. Lysates were pre-cleared at 800×g for 10 min.

### Western Blot Analysis and Antibodies

Protein concentrations were measured using BioRad Dc Protein Reagent. Proteins were separated on 4–15% gradient SDS-polyacrylamide gels (BioRad) under reducing conditions. Proteins were transferred to polyvinylidene fluoride membrane (Millipore) in Tris-glycine buffer pH 7.5 containing 10% methanol. Filters were blocked at RT in 5% non-fat dry milk in TBST [0.1 M TrisHCl pH 7.5, 0.15 M NaCl, and 0.1% Tween-20 (Sigma)]. Incubation with primary antibodies was overnight at 4°C. After TBST wash (4×5 min), appropriate secondary HRP-conjugated antibodies were applied for 90 min at RT. Filters were washed, as above, then developed with ECL (Pierce) and exposed to Highblot CL autoradiography film. Films were scanned using an Epson V600 scanner. Densitometry was performed using NIH ImageJ 1.44f software [http://rsb.info.nih.gov/ij].

Antibodies used were: anti-LamR 1∶1000 (H-2, mouse mAb, 74515 Santa Cruz) in 5% milk; anti-Sup35 1:1000 in PBS (BE4 mouse mAb, [52], a gift from Susan Liebman to ILD); anti-GFP 1∶1000 (Invitrogen rabbit pAb, A11122) in 5% milk; anti-yeast hexokinase-HRP 1∶1000 (Abcam, ab34588) in 5% milk.

### Coimmunoprecipitation

Immunoprecipitation was performed using magnetic, Protein G, Dynabeads (Dynal, Invitrogen) according to manufacturer’s protocol. Anti-GFP antibody (10 µg) was adsorbed to 50 µl bead slurry. Control beads were prepared with 10 µg rabbit IgG. Yeast cell lysates (500 µg in 200 µl lysis buffer) were added to prepared antibody-bound beads and rotated overnight at 4°C. Beads were removed magnetically, and supernatant removed as unbound (flow thru, FT) fraction. Beads were washed with 500 µl PBS with 0.02% Tween-20 (PBST), transferred to a new tube with PBST and washed an additional 2×. One ml TBST was added to separated beads. Beads were transferred to a new tube and washed 3× in TBST. Bound protein was removed by incubation of separated beads at 70°C, 10 min in 1× SDS PAGE buffer (50 µl). Eluted protein (25 µl) and 20 µg total cell lysate and FT fraction were separated on SDS PAGE gels and analyzed by western blot.

### Fluorescence Microscopy

Cultures of yeast transformants, grown at 30°C in plasmid selective media supplemented with 25 µM CuSO_4_ from 0.02 OD 600 nm to late-log (48 hours) were viewed with the Plan Fluor 100x/1.3 oil DIC lens of a Nikon TE-2000E fluorescent microscope using a 488 nm_ex_, 507 nm_em_ filter for GFP and a 589 nm_ex_ 615 nm_em_ filter for mCherry. Images were captured with a Nikon CoolSnap EZ camera and processed with NIS Elements V2.3 software.

### High Speed Centrifugation Analysis

Lysates for centrifugation assays [36] were prepared as described above except that RNAse A (400 µg/ml) was added to yeast lysate buffer to disrupt ribosomes and lysates were precleared at 8000×g, 3 min [53]. Approximately 200 µl lysate was spun, 30 min, at 100,000×g in a Beckman TLA 120.1 rotor (Beckman Optima TLX ultracentrifuge). Supernatants were carefully removed and pellets resuspended in equal volume (200 µl) of lysis buffer. Equal amounts (20 µg) of total, supernatant and pellet protein were analyzed by western blot.

## Results

### Expression of Human LamR in Yeast Cell

**Figure 1:**
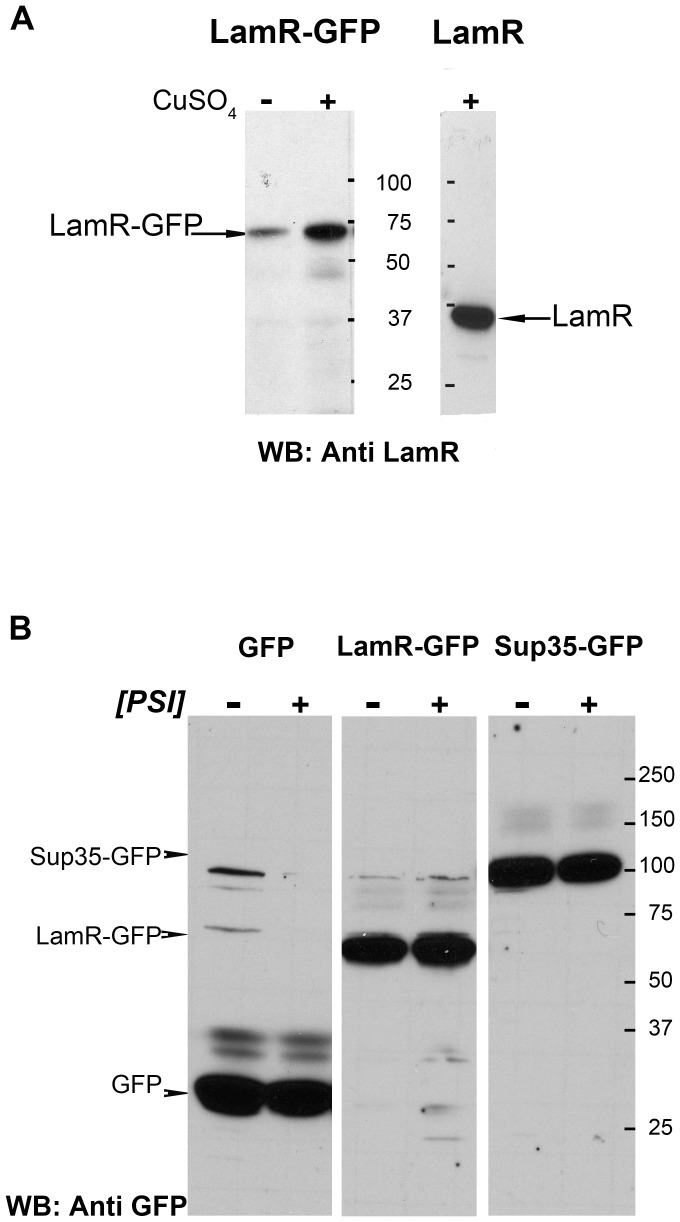
Human LamR is expressed in yeast cells. A. LamR protein (right panel) or LamR-GFP fusion (left panel) expressed in the [*PSI* ^+^][*PIN^+^*] yeast prion strain grown in synthetic media either supplemented with 25 µM CuSO_4_ (right panel and lane 2 of left panel) or containing no excess copper (lane1 of left panel). B. Expression of GPF (27 kDa), and LamR-GFP and Sup35-GFP fusion proteins (64 kDa and 104 kDa, respectively) in [*psi* ^−^][*PIN^+^*] (−) and [*PSI^+^*][*PIN^+^*] (+) yeast strains. Anti-LamR (A) and anti-GFP (B) antibodies were used to detect LamR expression in yeast lysates (25 ug). Numbers in the middle (A) and right (B) refer to protein size markers (kDa). Similar expression levels were observed in [*pin* ^−^] strains (not shown).

The human LamR was expressed under the control of the copper inducible *CUP1* promoter from a low-copy yeast plasmid. Both untagged and C-terminal GFP fusion constructs were made. The size of the expressed proteins was as expected, ∼37 and 64 kDa, respectively, and expression levels were similar for the untagged LamR and the GFP LamR fusion proteins (Fig. 1A, right panel and left panel, respectively). The GFP fusion construct was utilized to enable intracellular visualization of LamR and provide an epitope for immunoprecipitation.

Figure 1A (left panel) shows that the *CUP1* promoter induction, in the presence of 25 µM CuSO_4_, enhanced expression of the 64 kDa LamR-GFP fusion protein compared to basal level expression observed in the cells grown in media with no excess CuSO_4_. The right panel shows expression of the untagged LamR grown in the presence of 25 µM CuSO_4_. While the mouse monoclonal LamR antibody H-2, raised against a polypeptide including amino acids 110–150 of the LamR, was able to recognize the human LamR protein in yeast cell lysates, the antibody does not react with any endogenous protein in the yeast extract. Specifically, an expected 30 kDa band for the orthologous RPS0 yeast ribosomal protein is not observed on western blots (Fig. 1A). Moreover, extract from untransformed yeast cells showed no reacting protein bands (not shown). Apparently, the epitope recognized by the LamR H-2 antibody is absent in RPS0 or is not strongly reactive with the H2 antibody.

When plasmids expressing GFP, LamR-GFP or Sup35-GFP, were transformed into yeast strains that either lacked or contained the [*PSI* ^+^] prion, the GFP, LamR-GFP and Sup35-GFP proteins were expressed at relatively equivalent levels in [*psi* ^−^][*PIN^+^*] and [*PSI^+^*][*PIN^+^*] yeast strains (Fig. 1B).

### LamR Interacts with Yeast Sup35 Protein

**Figure 2:**
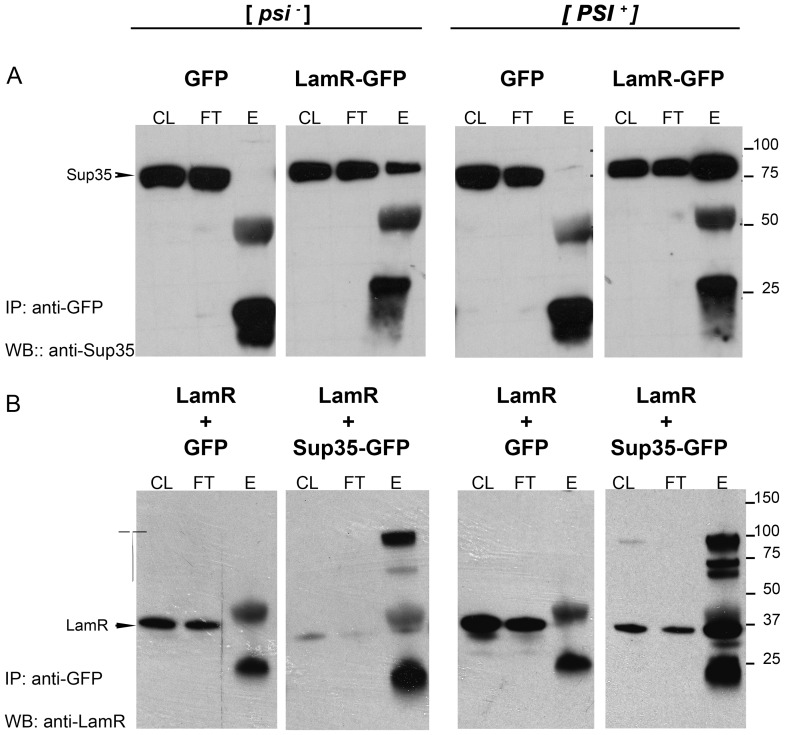
Human LamR co-immunoprecipitates with yeast Sup35 protein. Yeast cell lysates (500 ug) from transformants of [*psi* ^−^][*PIN^+^*] and [*PSI^+^*][*PIN^+^*] strains carrying (A) pCUP1-GFP or pCUP1-LamR::GFP and (B) wild type pCUP1-LamR together with either pCUP-GFP or pCUP-SUP35::GFP were precipitated with anti-GFP antibody. Total eluted proteins (E) were compared with 20 ug cell lysate (CL) and unbound (flow thru, FT) samples; eluted samples represent 12× the amount of CL and FT. Western blots were probed with anti-Sup35 antibody (A) or anti-LamR antibody (B). Numbers on the right (A and B) refer to protein size markers (kDa). High molecular weight LamR bands are observed from 60–100 k Da in the presence of Sup35-GFP (B, panels 2 and 4). The 50 kDa and 25 kDa bands in A and B are the heavy and light chain IgG bands, respectively. Shown are representative experiments; similar results have been obtained for at least three independent experiments.

To assess the interaction of the human LamR with yeast Sup35 monomers or Sup35 prion aggregates/oligomers, co-immunoprecipitation experiments were performed. Lysates of CuSO_4_-induced cells were incubated with protein G-linked magnetic beads bound with GFP antibody. Figure 2 shows western blots of GFP antibody-precipitated lysate samples that were probed with antibody to Sup35 (A) or LamR (B). In lysates expressing LamR-GFP (Fig. 2A, panels 2 and 4), 76 kDa Sup35 protein bands appear in the eluted fractions of both [*psi* ^−^] and [*PSI* ^+^] yeast strains, but the relative amount of pulled-down Sup35 is significantly higher in the [*PSI* ^+^] lysates. Lysate fractions eluted from yeast that expressed only GFP protein did not show a 76 kDa Sup35 protein band indicating a specific interaction between the LamR and Sup35 proteins (Fig. 2A, panels 1 and 3). Also, control beads bound with mouse IgG did not bind Sup35 (not shown).

**Figure 3:**
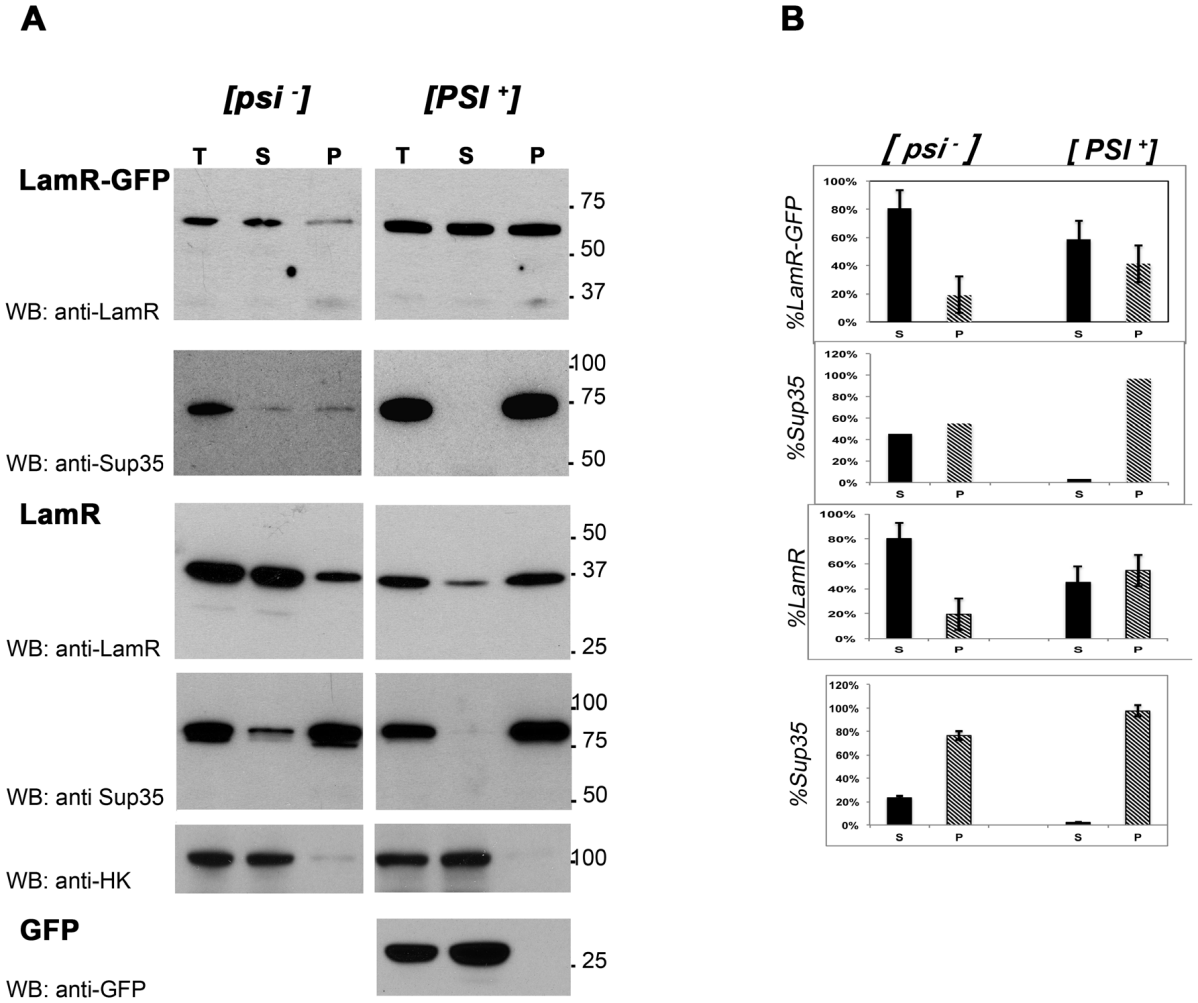
Centrifugation assay demonstrates aggregation of exogenous human LamR in the presence of the [*PSI* ^+^] prion. (A) Total lysate (T), supernatant (S), and resuspended pellet (P) (20 µg per sample) of pCUP1-LamR::GFP and pCUP1-LamR transformants of yeast [*psi* ^−^][*pin* ^−^] and [*PSI^+^*][*PIN^+^*] strains were analyzed by western blot using the indicated antibodies. pCUP1-GFP was expressed in the [*PSI* ^+^] strain as a control (bottom panel), and anti-yeast hexokinase antibody (anti-HK) was used to ensure pellets were free of cytoplasmic proteins (fifth panel from top). Shown are representative experiments out of 3 independent experiments. (B) Corresponding densitometric quantitation of percent distribution between supernatant and pellet fractions was determined from three independent experiments. Bars show standard error of the mean.

Although a significantly lesser amount of Sup35 eluted from LamR beads in the [*psi* ^−^] strain, the elution of small amounts of Sup35, as opposed to complete absence of Sup35 in eluates, may be due to an interaction of LamR with the monomeric Sup35, or due to the presence of insignificant amounts non-heritable Sup35 oligomers. Indeed, some amounts of aggregated Sup35 are always detected in centrifugation assays in [*psi* ^−^] strains (see Fig. 3).

Co-expression of wild type LamR with GFP or Sup35-GFP showed similar results (Fig. 2B). Expression of LamR with GFP protein alone did not result in the elution of a 37 kDa LamR band from anti-GFP coated beads in either [*psi* ^−^] or [*PSI* ^+^] yeast lysates (panels 1 and 3). In [*psi* ^−^] yeast co-expressing LamR with Sup35-GFP very small amounts of 37 kDa LamR were observed in anti-GFP eluates, but a very strong band specifically recognized by anti-LamR appeared at approximately 100 kDa. [*PSI* ^+^] yeast lysates co-expressing LamR with Sup35-GFP contained significantly higher amounts of 37 kDa LamR (Fig. 2B) and even greater amounts of higher molecular weight bands were also observed. Filters, completely stripped of LamR antibody, and reprobed with Sup35 antibody showed coincident higher molecular weight bands (not shown).

The co-immunoprecipitation experiments reveal an interaction between LamR and Sup35, the exact nature of which is not yet clear. Associations may occur between monomeric proteins, oligomers or aggregates. Presence of Sup35 and LamR together in higher molecular weight bands may be caused by anomalous migration of proteins in SDS PAGE gel due to aggregation.

### Co-distribution of LamR with Sup35 Prion Protein

Centrifugation analysis provides another method to examine the aggregation of LamR in the presence of the Sup35-based prion. High-speed centrifugation sediments prion aggregates whereas non-aggregated forms of the proteins tend to remain in the supernatant [36]. Total yeast cell lysates, 100,000×g pellets and soluble supernatant fractions were analyzed by western blot. In this experiment, both pCUP1-LamR::GFP and pCUP1-LamR transformants were used, and supernatant and pellet fractions were probed for the exogenous human LamR and the endogenous Sup35 proteins. To ensure that unbroken cells or supernatant lysate did not contaminate the pellet fractions, membranes were probed with antibody to yeast hexokinase 1, which is located only in the cytoplasm (Fig. 3A).

In [*PSI* ^+^] cell lysates, whether LamR or LamR-GFP was expressed, almost all Sup35 and >40% of LamR were detected in the pellet fraction, indicative of their aggregated state. Conversely, in [*psi* ^−^] cells, the distribution of both Sup35 and LamR was shifted towards the soluble fraction. The ratio of aggregated LamR to soluble LamR was reversed, with the vast majority of LamR detected in the supernatant (∼80%). This further indicates that LamR becomes insoluble in the presence of the [*PSI* ^+^] prions.

To further exclude the possibility that GFP contributed to LamR aggregation, analysis of pCUP1-GFP transformants showed that the GFP protein did not produce aggregates regardless of the presence of [*PSI* ^+^] (Fig. 3A and not shown).

In summary, detection of LamR in the high-speed centrifugal pellets of the [*PSI* ^+^] lysates supports an association of LamR with aggregated Sup35 protein.

### LamR-GFP Forms Fluorescent Foci in [PSI^+^] Yeast Strains

**Figure 4:**
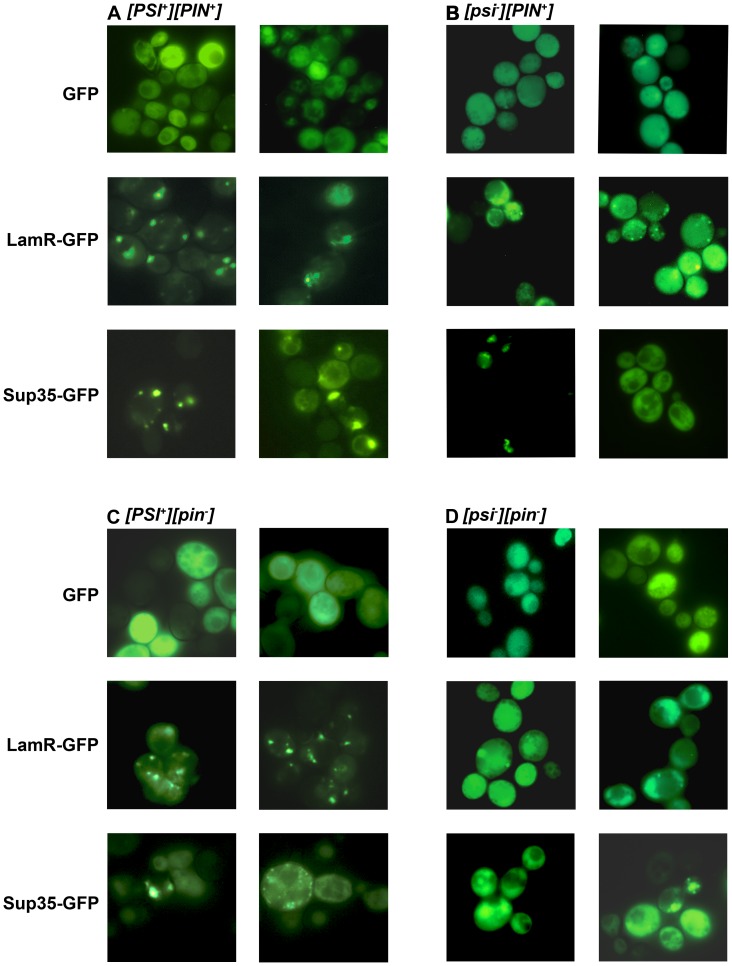
Immunofluorescence of LamR-GFP and Sup35-GFP fusion proteins reveals aggregation of LamR in [*PSI* ^+^] cells. Late-log cultures of (A) [*PSI^+^*][*PIN^+^*], (B) [*PSI^+^*][*pin* ^−^], (C) [*psi* ^−^][*PIN^+^*] and (D) [*psi* ^−^ ][*pin* ^−^] were examined with 100× oil immersion lens of a fluorescent microscope using a 488_ex_, 507_em_ filter. Representative images from two independent transformants of each yeast strain are displayed.

As the co-immunoprecipitation and co-distribution experiments indicated the [*PSI* ^+^] prion-dependent interaction of LamR and Sup35, we examined whether, like Sup35, LamR forms visible cytoplasmic aggregates in yeast cells. The distribution of GFP, LamR-GFP and Sup35-GFP was examined in [*PSI* ^+^] and [*psi* ^−^] cells. Figure 4A displays representative images of yeast cells from two independent transformants of the [*PSI* ^+^] strain. As expected, yeast cells expressing GFP showed relatively even, diffuse, cytoplasmic distribution of fluorescence. Also, as shown before [36], in [*PSI* ^+^] cells that expressed Sup35-GFP, Sup35 was detected predominantly in bright fluorescent foci with hardly any protein visible in the cytoplasm. The expression pattern of LamR-GFP strikingly resembled that of Sup35: in most cells LamR was seen in bright foci with very little non-aggregated protein.

In [*psi* ^−^][*PIN^+^*] cells (Fig. 4 B) both Sup35 and LamR-GFP were usually distributed evenly in the cytoplasm. However, in some cells both Sup35-GFP and LamR formed [*PSI* ^+^] like aggregates. The formation of [*PSI* ^+^] aggregates following overexpression of the Sup35-GFP construct was expected because the strain used in this experiment carries another prion, [*PIN^+^*]. The presence of the [*PIN^+^*] prion, which is a self-propagating state of the Rnq1 protein [46], [47], [54], [55], allows for the *de novo* [*PSI* ^+^] formation upon overexpression of the Sup35 protein [46], [47], [54], apparently due to the direct seeding of the [*PSI* ^+^] prion by the pre-existing [*PIN^+^*] prion aggregates [54], [56], [57], [58]. The LamR aggregates in these [*psi* ^−^][*PIN^+^*] cells could result from either co-aggregating with the newly forming [*PSI* ^+^] or from interaction of LamR with [*PIN^+^*].

To distinguish between these possibilities, we employed [*pin* ^−^] variants of the same strains, [*PSI^+^*][*pin^–^*] (Fig. 4C) and [*psi* ^−^][*pin* ^−^] (Fig. 4D). In [*PSI^+^*][*pin^–^*] transformants, aggregation patterns of all proteins, GFP, Sup35-GFP and LamR-GFP were indistinguishable from [*PSI^+^*][*PIN^+^*] indicating that [*PIN^+^*] did not determine the LamR aggregation (Fig. 4C). In [*psi* ^−^][*pin* ^−^] cells both Sup35 and LamR-GFP were evenly distributed in the cytoplasm (Fig. 4D). As new [*PSI* ^+^] formation does not occur when Sup35 is overproduced in the absence of [*PIN^+^*] [47], the fact that LamR-GFP was also non-clustered in [*pin* ^−^][*psi* ^−^] cells strongly indicates that LamR aggregation in [*PSI^+^*][*PIN^+^*] (Fig. 4A) and [*PSI* ^+^][*pin* ^−^] cells (Fig. 4C) was driven by the [*PSI* ^+^I] prion, and suggest that LamR binds to the pre-existing and newly forming [*PSI* ^+^] prion.

### Sup35-GFP and LamR-mCherry Co-localize with [PSI^+^] or Newly Forming [PSI^+^]

**Figure 5:**
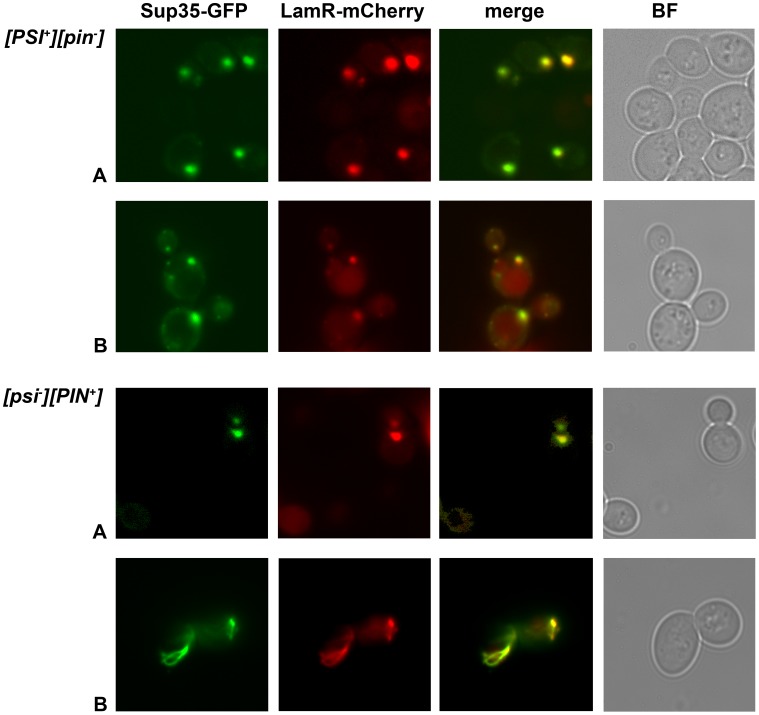
LamR co-localizes with visible Sup35 prion aggregates in yeast cells. pSUP35-GFP and pLamR-mCherry were co-expressed in weak [*PSI^+^*][*pin* ^−^] and [*psi* ^−^][*PIN^+^*] cells. Panels show GFP and mCherry fluorescence and their merged images. Brightfield (BF) images of the cells are shown in the far right panel. Two sets of images (A and B) are shown from independent transformants. Images were taken using a100× oil immersion lens. The images were visualized and merged using Adobe photoshop CS4.

Analysis of fluorescent aggregates in cells co-expressing Sup35-GFP and LamR-mCherry clearly reveals the [*PSI^+^*]-dependent interaction between the Sup35 and LamR proteins. Overlapping Sup35 and LamR punctate foci were observed in cells of the weak [*PSI^+^*][*pin* ^−^] strain transformed with pCUP1-SUP35::GFP and pCUP1-LamR::mCherry (Figure 5, two top rows, A and B refer to two independent transformants). Sup35 and LamR foci could also be found in strong prion yeast cells, however, the cytotoxicity associated with over-expression of Sup35 (pCUP1-SUP35::GFP) in the strong [*PSI* ^+^] strains [59] made it difficult to detect and image aggregate-containing cells (not shown). Also, as expected, if the presence of LamR foci were coupled with the [*PSI* ^+^] prion, and consistent with observations described in Figure 4D, fluorescently-labeled Sup35 and LamR were evenly dispersed throughout the cytoplasm of [*psi* ^−^][*pin* ^−^] yeast transformants (not shown).

We also followed localization of LamR and Sup35 in [*psi^–^*][*PIN^+^*] cells where the [*PSI* ^+^] prion is forming *de novo* (see Figure 4B above). The newly generated prions can take the form of punctate dots, ring-like or branched ring structures [49]. The two bottom panels of Figure 5 show cell clusters from two independent [*psi^–^*][*PIN^+^*] transformants. The first cluster (A) contains dot aggregates for Sup35, indicative of heritable [*PSI^+^*], and the second cluster – a branched ring (B), which is a non-mature form of [*PSI^+^*]. In both cases Sup35 aggregates co-localize with LamR-mCherry visible aggregates. These results suggest that LamR may interact with different prion-like conformers of Sup35. Co-localization is observed in both mother and daughter cells, indicative of a heritable transmission of LamR with [*PSI^+^*], although re-association cannot be excluded by these experiments.

### LamR-GFP Expression does not Change the [PSI] Status of Yeast Cells

Previous experimental evidence shows that interactions of prion-forming proteins with various cellular proteins may result in either *de novo* appearance or loss of pre-existing prions; this is true for both interactions between two different prion forming proteins (reviewed in [60]), and between prion-forming proteins and proteins that are not amyloidogenic themselves. Such non-amyloidogenic proteins include various chaperones (reviewed in [61]) or overexpressed interacting partners of prion-forming proteins, like Sup45, which forms a translation termination complex with soluble Sup35 [61], [62]. We tested if LamR expression can cure [*PSI* ^+^] in [*PSI* ^+^] strains or promote [*PSI* ^+^] formation in [*psi* ^−^] cells. Using standard genetic assays to examine the propagation or loss of Sup35 prions [63], [64], [65], no effect was observed when pCUP1-LamR or pCUP1-LamR::GFP were expressed in [*PSI^+^*][*PIN^+^*], [*psi* ^−^][*pin* ^−^], [*PSI* ^+^][*pin* ^−^] or [*psi* ^−^][*PIN^+^*] yeast strains for over 30 replicative generations (not shown).

## Discussion

The data presented show an *in* *vivo* interaction between LamR and Sup35 protein when Sup35 is present in the prion [*PSI* ^+^] state. 1) In co-immunoprecipitation assays, Sup35 protein is pulled down with LamR-GFP from yeast cell lysates and, conversely, LamR is pulled down with Sup35-GFP). 2) In centrifugation assays, in [*PSI* ^+^] cell lysates, LamR was shifted to pellet fractions together with insoluble Sup35 protein. 3) Aggregation patterns of LamR and Sup35 were strikingly similar, with LamR-GFP forming punctate fluorescent foci in [*PSI* ^+^] cells and in cultures where [*PSI* ^+^] formation is possible. Furthermore, visible LamR-mCherry co-localized with both pre-existing and newly forming [*PSI^+^*]. Although the nature of the interaction between LamR and Sup35 prion protein has not been elucidated, the results indicate the adherence of LamR to [*PSI* ^+^] prion aggregates, as opposed to, interference or enhancement of the amyloidogenic process.

As part of the protein translation machinery, the yeast and human orthologs of LamR and Sup35, respectively, are highly homologous. The homology, however, is not universally strong throughout the proteins. For both Sup35 and LamR there is an ancient, highly conserved part of the protein and a more recently acquired variable extension. For example, the yeast ortholog of LamR, RPS0, has 252 amino acid residues compared with LamR (RPSA), which contains 295 residues. The additional amino acid residues of mammalian RPSA comprise a C-terminal domain that is thought to have evolved with the gain of the laminin binding function as organisms became multicellular [7]. Likewise, yeast Sup35 (eRF3) is a member of a protein family including ancient EF-Tu/eEF1A elongation factors [35]. The Sup35 region that is homologous with EF-Tu/eEF1A encompasses the GTP- and aminoacyl-tRNA-binding sites, is highly conserved and sufficient for viability and translation termination; respective regions of yeast and human proteins are 57% identical and 75% similar. On the other hand, the ∼200 amino acid long N-terminal extension, present only in Sup35/eRF3 proteins, is more variable. The exact role of this N-terminal extension is not known. In yeast it encompasses the Q/N-rich amyloidogenic region at the extreme N-terminus, which is essential for [*PSI* ^+^] formation and maintenance [35], [40], [66]. While mammalian eRF factors lack the Q/N-rich region, other segments of their N-terminal extension share similar amino acid composition with Sup35/eRF3, suggesting the possibility of functional conservation.

Although interaction of LamR (RPSA) and Sup35 as parts of the ribosomal complex is an obvious hypothesis, our results do not support an interaction that is dependent exclusively upon their ribosomal functions. Indeed, in this case, the interaction is expected to be as efficient when Sup35 is in a soluble non-prion state, whereas, our data suggest that interaction is facilitated by the aggregated protein. In addition, the fact that human eRF3 proteins have not been found to be in complex with LamR in a stringent proteomic study of human LamR binding proteins indicates that interaction does not occur within the framework of a major and constitutive cellular process involving a considerable fraction of each protein [67].

Our data indicate, rather, that interaction between LamR and Sup35 is directed by their newly gained functions, implicating their more recently acquired domains. Acquisition of an extended C-terminus by LamR is thought to be important for the cell surface localization of LamR, its external position allowing for extracellular interactions [7], [20]. The importance of this functional role is reflected by the very high degree of conservation in vertebrates throughout the entire LamR protein sequence [7]. Positioned externally, the LamR C-terminus can bind with laminin and serve as a receptor for various molecules, including PrP^C^ and PrP^Sc^, as cited in the introduction.

The ability of LamR to bind PrP^Sc^ [23], [24] has led us to hypothesize that the LamR may have an affinity for structures characteristic of prions and amyloidogenic proteins. Indeed, while there is no evidence that formation of laminin-1, first used to isolate LamR, involves typical amyloid, it contains a fibrous, coiled-coil, α-helical domain that forms a network in the extracellular matrix [68], [69]. Furthermore, amyloidogenic sequences have been identified in laminin-1. Peptides of these sequences form amyloid fibers *in* *vitro* and can presumably form *in* *vivo* when laminin-1 is fragmented or unstructured while undergoing conformational transformations [70]. Analysis indicates that the extracellular C-terminal domain of LamR is a disordered structure [71]. It is increasingly apparent that disordered domains are common among proteins that form multiple protein-protein interactions [72], [73], and may be both involved with specific binding or engage in protein-protein contacts in a less specific manner. Different regions or conformations of the LamR protein may modulate its interaction with human PrP^C^ *vs* PrP^Sc^. While specific binding sites have been identified, on each protein, for interaction between LamR and PrP^C^ [22], LamR binding with PrP^Sc^ has not been defined.

Significant experimental evidence suggests that both LamR and Sup35-based prions are associated with the actin cytoskeleton, and thus it is plausible that the actin cytoskeleton can mediate LamR and Sup35/[*PSI* ^+^] interaction. Components of the actin cortical cytoskeleton have been shown to interact with the prion domain of Sup35. This association promotes *de novo* [*PSI* ^+^] prion formation and aggregation [74]. LamR has also been shown to interact with the cytoskeletal network within mammalian cells [8], [75], [76], [77], [78]. Specifically, co-localization has been shown with actin filaments *in* *vivo* [8], [78] and *in* *vitro* [78] and interaction with microfilaments is important for cell adhesion and motility. Ganusova *et* *al.* have proposed a model whereby the yeast actin cytoskeleton acts as a scaffold for the amyloid-based aggregation of misfolded proteins, reducing the toxicity of misfolded proteins by sequestration from the cytosol [74]. The cytoskeleton may also serve as a location for protein refolding. Chaperone proteins that contribute to [*PSI* ^+^] propagation [61], [74] have been found at the cytoskeleton [74]. In this regard, it is noteworthy that LamR amino acid residues 1–120 share some homology with the Hsp70 chaperone protein [79].

In conclusion, our findings reveal the propensity of LamR to interact with different prion-forming proteins and raise the possibility that LamR interaction with mammalian prion protein occurs not only in the capacity of the PrP^C^ receptor, but is implicated in either prion infectivity or prevention of prion infection through a structural affinity for PrP^Sc^. Utilization of the yeast assay system provides a safe and easily manipulated system for further study of LamR binding to prions and amyloids. Structure guided mutagenesis has been used to delineate the laminin-1 binding site of LamR [80]. Similarly, mutagenesis experiments can be designed to probe the interaction of LamR with Sup35 and other amyloid-like proteins. It is hoped that such studies will facilitate an understanding of the multifunctional interactions of the LamR protein.
